# Actomyosin contractility and buckling of microtubules in nucleation, growth and disassembling of focal adhesions

**DOI:** 10.1007/s10237-022-01584-3

**Published:** 2022-05-25

**Authors:** S. Palumbo, E. Benvenuti, M. Fraldi

**Affiliations:** 1 Department of Structures for Engineering and Architecture, University of Napoli ”Federico II”, Napoli, Italy; 2 Department of Engineering, University of Ferrara, Ferrara, Italy

**Keywords:** Focal adhesions growth, Cellular tensegrity, Cell mechanobiology, Nonlinear elasticity

## Abstract

Building up and maintenance of cytoskeletal structure in living cells are force-dependent processes involving a dynamic chain of polymerization and depolymerization events, which are also at the basis of cells’ remodelling and locomotion. All these phenomena develop by establishing cell–matrix interfaces made of protein complexes, known as focal adhesions, which govern mechanosensing and mechanotransduction mechanisms mediated by stress transmission between cell interior and external environment. Within this framework, by starting from a work by Cao et al. (Biophys J 109:1807–1817, 2015), we here investigate the role played by actomyosin contractility of stress fibres in nucleation, growth and disassembling of focal adhesions. In particular, we propose a tensegrity model of an adherent cell incorporating nonlinear elasticity and unstable behaviours, which provides a new kinematical interpretation of cellular contractile forces and describes how stress fibres, microtubules and adhesion plaques interact mechanobiologically. The results confirm some experimental evidences and suggest how the actomyosin contraction level could be exploited by cells to actively control their adhesion, eventually triggering cytoskeleton reconfigurations and migration processes observed in both physiological conditions and diseases.

**Supplementary Information:**

The online version contains supplementary material available at 10.1007/s10237-022-01584-3.

## Introduction

Adhesion is at the basis of a number of cells’ physiological functions, such as motility, differentiation, proliferation and maintenance of tissues’ homeostasis, as well as of pathological processes like wound healing and tumour invasion. The development of these phenomena in fact relies on—and in turn affects—mechanosensing and mechanotransduction mechanisms through which the living cell can sense the physical stimuli coming from its surroundings and respond to them by activating cascades of bio-chemo-mechanical events (Bershadsky et al. 2003; Discher Dennis et al. 2005; Janmey et al. 2020; Schwarz and Safran 2013). To do this, cells are equipped with an internal biopolymeric structure—the cytoskeleton (CSK)—that gives them shape, stability and elastic stiffness by essentially obeying the tensegrity self-equilibrium principle, that is by guaranteeing the mechanical balance between pre-tension travelling along its actin constituents—namely actomyosin microfilaments and stress fibres (SFs)—and the pre-compression born by microtubules (MTs) (Ingber et al. 2014; Fraldi et al. 2019; Palumbo et al. 2018). In this way, the CSK constitutes a preferential path for mechanical forces transmission both among the different cellular districts and between the cell inner and the extra-cellular matrix (ECM) (Wang et al. 2009). In the latter case, forces exchange is mediated by the presence of molecular assemblies comprising structural and signalling proteins that connect the CSK to the ECM by crossing the cellular membrane, known as focal adhesions (FAs) (Geiger et al. 2001, 2009). FAs’ loci essentially consist of an agglomerate of trans-plasma-membrane integrin receptors that bind, through the extra-cellular domain, to specific ligands within the ECM and, at the intracellular end, to actin sites of the CSK via adapter proteins, such as talin, vinculin and paxillin, which form a multi-layer adhesion plaque (Kanchanawong et al. 2010).

**Fig. 1:**
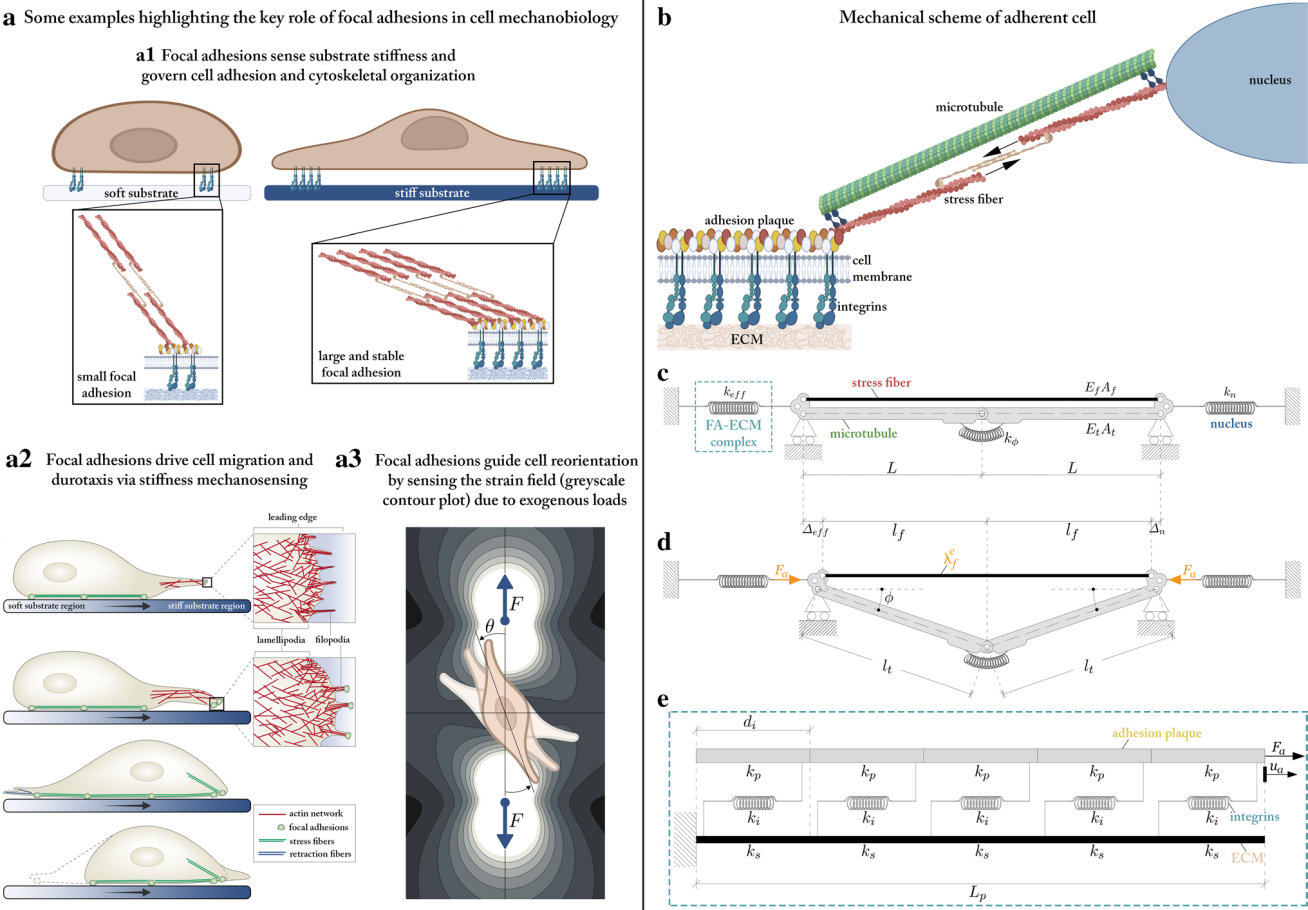
**a** A synoptic scheme reporting some key cellular processes mediated by the mechanosensing and mechanotransduction functions of FAs, such as: **a1** differential adhesion over substrates with different deformability (Discher et al. 2005); **a2** directional migration from soft to stiff regions of an elastic substrate, propelled by actin-dependent protrusions of the cell leading edge, i.e. filopodia and lamellipodia (the related image has been re-adapted from the work by Mattila and Lappalainen (2008)); **a3** cell reorientation under the action of exogenous loads, along optimal directions depending on the mechanical properties of the underlying medium and on the features of the applied forces, e.g. on their static or dynamic nature (Palumbo et al. 2021). **b** Sketch of an adherent cell comprising the nucleus, the cytosketetal compartment, made of an actomyosin SF and a MT, and the FA complex, comprising the adhesion plaque and integrin receptors binding to the ECM by crossing the cell membrane. **c** Mechanical model of the adherent cell in its stress-free reference state and **d** in its current configuration, deformed—with possible MT buckling—as a consequence of the activation of actomyosin contraction in the SF. **e** Focus on the structural scheme adopted for the FA-ECM complex, whose overall equivalent stiffness is given by $k_{eff}=F_a / u_a$ (borrowed from the work by Cao et al. (2015))

As a matter of fact, nucleation and maturation of stable FAs strongly depend on the mechanical forces that they bear (Balaban et al. 2001). Indeed, it has been experimentally demonstrated that cytoskeletal actomyosin contractility, actively generating tensile forces in both single actomyosin microfilaments and assembled SFs, regulates the molecular kinetics of FAs, its inhibition gradually leading to FAs’ disassembly (Wolfenson et al. 2011; Deshpande et al. 2008). In addition, several works have shown that, in the most of adherent cell types, FAs’ growth is enhanced by the localized application of external pulling loads (Riveline et al. 2001) as well as by the stiffening of the culture substrate (Prager-Khoutorsky et al. 2011; Trichet et al. 2012; Fusco et al. 2017), which in fact contribute to determine both their size and morphology. As a result, the stress-dependent continuous chain of polymerization and depolymerization events occurring at the cell-ECM junctions influences the cell mechanosensing and mechanotransduction functions. Indeed, by simultaneously exploiting FAs as mechanosensors and mechanotransducers, adherent cells are capable of probing the elastic properties of their microenvironment by anchoring and pulling on it as well as of translating the perceived stimuli into (still not completely known) biochemical pathways that can activate a number of intracellular responses and thus govern entire cellular processes. In particular, it is by now consolidated the observation that cells can remodulate their cytoskeletal organization as a function of the stiffness and of the stress/strain patterns that they sense (Deshpande et al. 2006), through FAs, from the ECM or from the synthetic substrate on which they lie, thus in turn adjusting their adhesion and contractility level, as sketched in Fig. 1a. This then leads, by way of example, to phenomena of cell spreading (Vernerey and Farsad 2014), migration and reorientation (Kim et al. 2012; Ben Amar et al. 2011; Deibler et al. 2011), including durotaxis (Lo et al. 2000; Trichet et al. 2012; Lazopoulos and Stamenović 2008) [27] and mechanotropism (Palumbo et al. 2021; Bischofs and Schwarz 2003), as well as affects important biological events, such as proliferation and differentiation (Discher et al. 2005; Nelson et al. 2005; Engler et al. 2006; Zhang et al. 2013).

In the light of these observations and, thus, of the necessity of tracing and explaining the emerging pivotal role played by FAs in cell mechanobiology, several theoretical models have been proposed in recent years (MacKay and Khadra 2020; Ronan et al. 2014; Deshpande et al. 2006; Vernerey and Akalp 2016; Deshpande et al. 2008; Vernerey and Farsad 2011, 2014; Schwarz and Safran 2013; Chen et al. 2015; Nicolas et al. 2004; Shemesh et al. 2005; He et al. 2014; Cao et al. 2015, 2017). For example, Nicolas et al. (2004) described the anisotropic growth of FAs in the direction of an applied force as a biochemical response to stress-induced integrins deformations in terms of addition and loss of molecules, also as a function of the ECM elastic properties. By looking at the same observation scale, Shemesh et al. (2005) thermodynamically studied the assembly/disassembly dynamics of a one-dimensional FA anchored to a rigid substrate under the action of a pulling load, in this way evidencing different regimes of the aggregate evolution depending on the selected ranges of the model parameters. On the other hand, Cao and co-workers (Cao et al. 2015) recently proposed a chemo-mechanical characterization of the development phases of the FAs of an adherent cell by modelling this overall as a discrete architecture comprising different (one-dimensional) structural elements representative of the FA-ECM complex, of the nucleus and of an interconnecting actomyosin SF. In this way, they showed that the initial size of the adhesion site and the stiffnesses of the ECM and of the nucleus directly influence the FA growth by cooperating with the contractility force originating in the SF-like constituent and a chemo-mechanical feedback effect.

In this framework, the present work aims to give a contribution for better understanding the role played by intracellular forces actively generated by SFs on the maturation and dismantling processes of cells’ adhesion loci. To do this, we start from the essential structural machinery adopted in the work by Cao et al. (2015) for depicting a single-cell adhering to the ECM via FAs and generalize it by including nonlinear elasticity and instability phenomena of the cytoskeletal components through a minimal tensegrity model. More in detail, we ascribe to an inelastic deformation aliquot of the SF-like hyperelastic element (activated chemically and realized by myosin motors) the origin of the tensile force that it bears, thus replacing the classical concept of intracellular contractile force with the equivalent one of inelastic (actomyosin-induced) contraction, having rather a kinematical nature. Furthermore, to take into account the necessary presence of MTs as CSK compression-bearing constituents, we introduce a MT-like one-dimensional component that interacts with the SF-like one by contributing to provide the overall mechanical equilibrium either by deforming hyperelastically or by undergoing elastic buckling. By integrating all these elements, we first show how the theoretical outcomes obtained by Cao et al. (2015), which confirmed laboratory evidences, can be all still retraced by employing the presented generalized approach. Then, by recovering buckling of MTs observed experimentally, we explore the effects of increasing levels of actomyosin contraction—producing large deformation regimes and, eventually, structural instabilities—on the magnitude of the mechanical forces standing on the cell FA and, in turn, on its growth rate, as a function of both FA starting size and ECM stiffness.

The work is organized as follows: Sect. 2 describes and explains the model here employed as mechanical equivalent of a single-cell anchoring to the ECM through FAs; Sect. 3 focuses on the thermodynamical derivation of the FAs growth rate as a function of the mechanical forces; finally, in Sect. 4, some sensitivity analyses and results of the model are illustrated and discussed.

## Mechanical modelling of an adherent single-cell

The mechanical scheme adopted in the following for modelling a cell adhering to the ECM is shown in Fig. 1b–e. Therein, a tensegrity system, comprising a single SF in parallel with a single MT (Palumbo et al. 2018), interconnects a spring, taking the role of the nucleus, to another elastic element, representative of the FA-ECM complex including the intracellular sub-membrane adhesion plaque and the trans-membrane integrins (Fig. 1b). Note that, without loss of generality, symmetry is assumed for the case under analysis and thus only one half of the cellular structure is represented in figure and modelled below. In particular, Fig. 1c shows the system in its stress-free (undeformed) reference state, while Fig. 1d provides a general actual configuration to which it can move as a consequence of the intracellular activation of the SF actomyosin contraction and of the resultant mechanical forces distributing throughout the structural components. Indeed, it is well known that *in vivo* SFs bear tensile forces (Deguchi et al. 2006; Vernerey and Farsad 2011), the origin of which mainly lies in the presence of myosin motors among their constituents (Ben Amar et al. 2018; Vuong-Brender et al. 2017; Vernerey and Akalp 2016). In particular, myosin, assisted by other cross-linking proteins (e.g. $\alpha$-actinin), transversely binds antiparallel actin microfilaments, thus forming bundles—the SFs—characterized by a regular structural arrangement throughout their length (Besser and Schwarz 2007). The activation of myosin heads through ATP hydrolysis then causes conformational changes that entail the mutual sliding between pairs of actin filaments (Stålhand and Holzapfel 2016). This would in turn produce an overall stress-free shortening of the single SF if it were free at the ends, but, actually, it results in the onset of tensile forces because of the elastic lateral constraints due to the interaction of the fibre with ECM, nucleus and MTs’ network. Such a phenomenon is generally taken into account in literature by modelling a SF as an elastic or viscoelastic element in parallel with a contractile component whose role is to impose a force of prescribed value, not constitutively related to any kinematics (Cao et al. 2015; Besser and Schwarz 2007; Cao et al. 2017). By starting from all these considerations and to better follow the actual mechanical response of the system induced by the actomyosin contraction by tracing the kinematical origin of tensile forces in SFs, we assume that SFs within cells behave as nonlinear elastic springs (Deguchi et al. 2006; De Tommasi et al. 2015; Puglisi et al. 2017) undergoing a deformation resulting from the superposition of the (incompatible) inelastic contraction that myosin motors would induce on the fibre if unconstrained and of an elastic elongation arising to ensure geometrical compatibility with external constraints that is with the elastic surroundings (Stålhand et al. 2016). Therefore, with reference to the equivalent structural scheme here adopted for an adherent cell at a general actual configuration (in Fig. 1d), the single SF bears a longitudinal stretch $\lambda _f$ given by:

2.1 $$\begin{aligned} \lambda _{f}=\lambda _{f}^{e} \lambda _{f}^{c}, \end{aligned}$$

obtained as multiplicative superposition of the inelastic contractile stretch $\lambda _f^c\in \left]0,1\right]$ and the elastic contribution $\lambda _f^e\in \left[ 1,+\infty \right[$ (Stålhand et al. 2016; Goriely 2017). As a consequence, the current length of the fibre can be expressed as:

2.2 $$\begin{aligned} 2l_{f}=\lambda _{f} \left( 2 L_{f} \right) =2\lambda _{f}^{e} \lambda _{f}^{c} L, \end{aligned}$$

where $L_f=L$ identifies the rest half-length of the fibre, assumed at an ideal stress-free reference configuration deriving from polymerization processes (Fig. 1c). Then, since the force arising within the fibre is purely related to the elastic amount of deformation—a dilation in the case at hand—it will be tensile, independently from the specific hyperelastic constitutive law. Furthermore, it is by now consolidated that tensile stresses borne by SFs and single actomyosin filaments are balanced by reaction forces of the ECM and by MTs, the latter behaving as compression-bearing elements according to the equilibrium rules governing tensegrity architectures (Stamenović and Ingber 2009; Fraldi et al. 2019; De Tommasi et al. 2015; Coughlin and Stamenović 1997). Therefore, to incorporate this key mechanical functioning principle, the cytoskeletal compartment linking the nucleus to a single FA is modelled through the simplest paradigm of soft tensegrity (Palumbo et al. 2018), which consists of a tensed cable, representative of the SF, in parallel with a compressed strut (the MT) that can contract hyperelastically and eventually buckle (Fraldi et al. 2021; Brodland and Gordon 1990; Brangwynne et al. 2006) thanks to the presence of a nonlinear elastic hinge. In particular, the axial contraction of the MT at a current configuration of the cell is assumed to be a purely elastic stretch $\lambda _t \in ]0,1]$, which determines its half-length $l_t$ as

2.3 $$\begin{aligned} l_{t}={\lambda _{t}}L_{t}={\lambda _{t}}L, \end{aligned}$$

with $L_t=L$ being the nominal MT half-length, which coincides with the one of the SF in order to ensure a stress-free condition at the reference configuration. Along with the axial stretch, the actual state of the MT is also defined by an inclination angle $\phi$, which can assume either finite or vanishing values—i.e. $\phi \in [0,\pi /2 [$—depending on whether the compressive force transmitted to it is high enough to produce instability or not, respectively. As a matter of fact, this relies on the myosin-guided level of inelastic contraction arising in the fibre as well as on the state in which the cell lies. In fact, in the case of a suspended cell, the SF-MT system would be self-equilibrated and the MT would be hence the sole structural element to sustain the tension coming from the SF. On the contrary, when a cell adheres through FAs to an elastic ground (the ECM or a functionalized substrate), the adhesion degree influences its configuration and the way in which stresses distribute, the balancing of forces resulting from the competition among MT, nucleus and the external anchor (Stamenović and Ingber 2009). In particular, to model the presence of these constituents, we here follow the same approach adopted by Cao et al. (2015), thus treating the nucleus and the FA-ECM complex as linear springs of stiffness $k_n$ and $k_{eff}$, respectively, placed at the extremities of the SF-MT system. The expression of the effective stiffness constant $k_{eff}$ is directly borrowed from the work by Cao et al. (2015) as:

2.4 $$\begin{aligned}&k_{eff}=\dfrac{d_i \left( k_p+k_s\right) }{L_c}\left[ \dfrac{L_p}{L_c}+2 {{\,\mathrm{csch}\,}}\left( \dfrac{L_p}{L_c}\right)+ \right. \nonumber \\&\quad \left. +\left( \dfrac{k_p}{k_s}+\dfrac{k_s}{k_p}\right) \coth \left( \dfrac{L_p}{L_c}\right) \right] ^{-1}, \quad L_c=d_i \sqrt{\dfrac{k_p k_s}{k_i \left( k_p + k_s \right) }}. \end{aligned}$$

Derivation of such expression involves the resolution of the linear elastic equilibrium problem with reference to the FA-ECM construct schematized in Fig. 1e, that is as a system made by an elastic fibre representing the adhesion plaque, free at one end and loaded by an axial force at the other extremity, connected to an elastic cantilever beam that simulates the ECM through equally spaced springs serving as integrin bonds. The value of $k_{eff}$ hence depends both on geometrical features, such as the length of the adhesion plaque $L_p$ and the average spacing $d_i$ between integrins, and on the mechanical properties of the single constituents, namely the stiffness $k_i$ of the integrins and the stiffnesses $k_p=E_p A_p/d_i$ and $k_s=E_s A_s/d_i$ characterizing, in the order, the adhesion plaque and the ECM/substrate, $E_p$ and $E_s$ being the corresponding Young moduli and $A_p$ and $A_s$ the nominal cross-sectional areas.

On these bases, by making reference to a general actual configuration of the overall system with buckled MT as in Fig. 1d, geometrical compatibility requires that:

2.5 $$\begin{aligned} l_f=l_t \cos \phi , \end{aligned}$$

which in turn, by virtue of Eqs. (2.1)–(2.3), allows to write:

2.6 $$\begin{aligned} \lambda _f^e=\dfrac{\lambda _t \cos \phi }{\lambda _f^c} \end{aligned}$$

Furthermore, geometrical arguments also impose:

2.7 $$\begin{aligned} \Delta =\Delta _{eff}+\Delta _n=2\left( L-l_f\right) =2L\left( 1-\lambda _t \cos \phi \right) , \end{aligned}$$

where $\Delta _{eff}= \alpha \Delta$ and $\Delta _n=\left( 1-\alpha \right) \Delta$, with $\alpha \in [0,1]$, are the displacements respectively undergone by the springs identifying the FA-ECM complex and the nucleus. It is worth noting that relationships (2.5)–(2.7) still hold true when the MT remains straight, i.e. when $\phi = 0$.

If both MT and SF obey an incompressible neo-Hookean law (Palumbo et al. 2018; Holzapfel 2000), the hyperelastic energies stored by each of their half tracts can be written as

2.8a $$\begin{aligned} \mathcal {U}_t&= \dfrac{E_t A_t L}{6} \left( \lambda _t^2 + \dfrac{2}{\lambda _t} -3 \right) , \end{aligned}$$

2.8b $$\begin{aligned} \mathcal {U}_f&= \dfrac{E_f A_f L \lambda _f^c}{6} \left[ \left( \lambda _f^e\right) ^2 + \dfrac{2}{\lambda _f^e} - 3 \right] \nonumber= \\ \quad&=\dfrac{E_f A_f L}{6} \left[ \dfrac{\left( \lambda _t \cos \phi \right) ^2}{\lambda _f^c} + \dfrac{2 \left( \lambda _f^c\right) ^2}{\lambda _t \cos \phi } - 3 \right] , \end{aligned}$$

$E_t$ and $E_f$ being the Young moduli of the MT and the SF, respectively, and $A_t$ and $A_f$ their transversal areas at rest. Also, in order to take into account the large angle variations that the rotational spring at the middle of the MT could experience at deviated configurations, it is here assumed that the stored nonlinear elastic energy has the form:

2.9 $$\begin{aligned} \mathcal {U}_{\phi }=-2 k_{\phi } \ln \bigg |\cos \left( \dfrac{\Delta \phi }{2} \right) \bigg |, \end{aligned}$$

so that the following constitutive relationship between the moment $M_{\phi }$ in the spring and the rotation $\Delta \phi =2\phi$ holds true:

2.10 $$\begin{aligned} M_{\phi }=k_{\phi } \tan \left( \dfrac{\Delta \phi }{2}\right) , \end{aligned}$$

where $k_{\phi }$ is the rotational stiffness constant. In particular, we here impose $k_{\phi }=\pi ^2 B_t/L$ in a way to guarantee that the critical compressive force inducing buckling of the MT when ideally inextensible and isolated at the extremities, coincides with the one calculated according to the Euler’s critical load formula (Euler 1744) in which $B_t$ is the MT bending stiffness. With reference to the latter, it is worth noting that its numerical value is chosen in the following (see Table 1) in a way to take into account the enhanced capability of *in vivo* MTs to resist compressive buckling, which is due to the lateral confinement provided by the surrounding cytosol and elastic network of other cytoskeletal filaments, namely actin microfilaments and intermediate filaments. This hence makes individual MTs’ bifurcation load increase orders of magnitude with respect to a laterally free case (Brangwynne et al. 2006; Brodland and Gordon 1990; Fraldi et al. 2019).

By virtue of this equivalence and all the above mentioned assumptions, the total potential energy associated to a buckled configuration of the system can be finally obtained as

2.11 $$\begin{aligned} \mathcal {E}=2 \left( \mathcal {U}_f + \mathcal {U}_t \right) + \mathcal {U}_{\phi } + \mathcal {U}_{eff} + \mathcal {U}_n, \end{aligned}$$

this coinciding with the sole internal elastic energy in case of absence of external forces acting on the cell and hence when the deformation process is activated and controlled by myosin motors via the inelastic contraction $\lambda _f^c$.

In Eq. (2.11), the energies $\mathcal {U}_{eff}$ and $\mathcal {U}_n$ are those respectively related to the FA-ECM complex and to the nucleus, which, because of linearity, are given by:

2.12a $$\begin{aligned} \mathcal {U}_{eff}&= \dfrac{1}{2} k_{eff} \Delta _{eff}^2=2 \, k_{eff} \, \alpha ^2 L^2 \left( 1-\lambda _t \cos \phi \right) ^2, \end{aligned}$$

2.12b $$\begin{aligned} \mathcal {U}_n&= \dfrac{1}{2} k_n \Delta _n^2=2 \, k_n \, \left( 1-\alpha \right) ^2 L^2 \left( 1-\lambda _t \cos \phi \right) ^2. \end{aligned}$$

Equilibrium equations can be then obtained by imposing stationarity conditions for $\mathcal {E}$ with respect to the Lagrangian parameters of the structure, e.g. the geometrical ratio $\alpha$, the axial stretch $\lambda _t$ of the MT and its inclination $\phi$, so that one has

2.13 $$\begin{aligned} \dfrac{\partial \mathcal {E}}{\partial \alpha }=\dfrac{\partial \mathcal {E}}{\partial \lambda _t}=\dfrac{\partial \mathcal {E}}{\partial \phi }=0, \end{aligned}$$

which, after some algebraic manipulations, leads to the following system of equations:

2.14a $$\begin{aligned}&\alpha \, k_{eff} - \left( 1- \alpha \right) k_n =0, \end{aligned}$$

2.14b $$\begin{aligned}&\left\{ \frac{2E_t A_t}{3} + \left\{ \frac{2E_f A_f}{3 \lambda _f^c }+ \right. \right. \\& \quad \left. \left. + 4 L \left[ \alpha ^2 \, k_{eff} + \left( 1- \alpha \right) ^2 k_n \right] {\frac{2E_f A_f}{3 \lambda _f^c }} \right\} \left( \cos \phi \right) ^2 \right\} \lambda _t^3 \\&\quad -4L\cos \phi \left[ \alpha ^2 \, k_{eff} + \left( 1- \alpha \right) ^2 k_n \right] \lambda _t^2 + \\&\quad -\frac{2}{3}\left[ E_t A_t +\frac{E_f A_f \left( \lambda _f^c \right) ^2}{\cos \phi }\right] =0, \end{aligned}$$

2.14c $$\begin{aligned}&\sin \phi \left\{ \frac{2 E_f A_f L}{3} \left[ \frac{\left( \lambda _f^c \right) ^2}{\lambda _t \left( \cos \phi \right) ^2} - \frac{\lambda _t^2 \cos \phi }{\lambda _f^c}\right] + \frac{2 k_{\phi }}{\cos \phi }+ \right. \\&\quad \left. +4 L^2 \lambda _t \left( 1-\lambda _t \cos \phi \right) \left[ \alpha ^2 \, k_{eff} + \left( 1- \alpha \right) ^2 k_n \right] {\frac{\left( \lambda _f^c \right) ^2}{\lambda _t \left( \cos \phi \right) ^2}} \right\} =0 . \end{aligned}$$

Eq. (2.14a) straightforwardly provides

2.15 $$\begin{aligned} \alpha =\dfrac{k_n}{k_{eff}+k_n}, \end{aligned}$$

the ratio between the elongations of the FA-ECM complex and of the nucleus depending, as expected, on the relative magnitudes of their equivalent stiffnesses. Then, resolution of the cubic Eq. (2.14b) with respect to the unknown $\lambda _t$, by resorting to the Cardano’s formula, provides:

2.16 $$\begin{aligned} \lambda _t=\frac{1}{3c_3}\left( \root 3 \of {-\frac{q}{2}-\sqrt{D}} + \root 3 \of {-\frac{q}{2}+\sqrt{D}} - c_2 \right) , \end{aligned}$$

where $D=q^2/4+p^3/27>0$, having set $q= 27 \, c_3^2 \, c_0 - 9 \, c_3 \, c_2 \, c_1 +2 \, c_2^3$ and $p =3 \left( 3 \, c_3\, c_1 - c_2^2 \right)$, the generic quantity $c_j$ indicating the coefficient of the Eq. (2.14b) multiplying the *j*-th power function of the unknown, with $j \in \left\{ 0,1,2,3 \right\}$. By finally employing such expression in the last Eq. (2.14c), one can find either the trivial solution $\phi =0$, corresponding to configurations in which the MT keeps straight, or a non-trivial one, describing a deviated state of MT, which results to be a function of the stretch $\lambda _f^c$ once assigned the geometrical and constitutive properties of all the constituents.

**Fig. 2:**
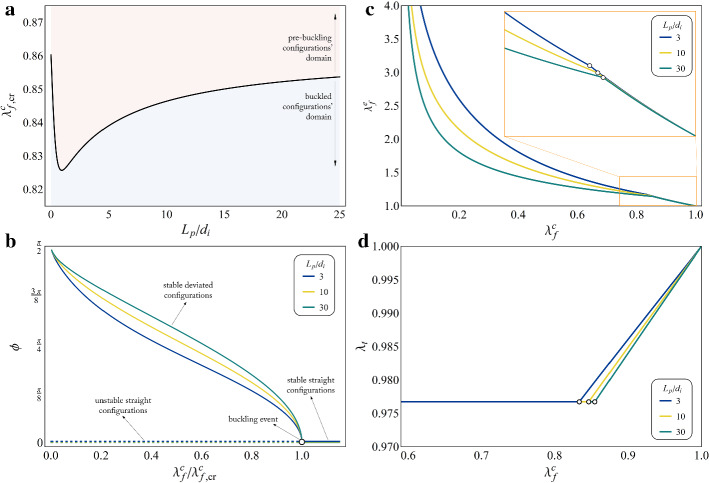
**a** Variation of $\lambda _{f,cr}^c$ as a function of the normalized plaque’s length $L_p/d_i$ and definition of pre- and post-buckling domains in the related phase space. **b** Equilibrium bifurcation path followed by the system for growing (from right to left) levels of SF inelastic contraction. The inclination angle $\phi$ of the MT is plotted as a function of the contractile stretch $\lambda _f^c$ normalized with respect to its critical value $\lambda _{f,cr}^c$, for three different lengths of the adhesion plaque, i.e. $L_p=3\, d_i,\, 10\, d_i,\, 30\, d_i$. Herein, solid tracts indicate (either straight or deviated) stable configurations while the dashed lines identify the unstable (straight) ones. **c** Elastic aliquot $\lambda _f^e$ of the stretch born by the SF and **d** purely elastic stretch $\lambda _t$ in the MT as functions of the actomyosin contraction $\lambda _f^c$. All the plots refer to values of the model’s parameters reported in Table 1, by in particular setting: $L=20\, \mu m$, $k_s=10 \, pN/nm$, $k_n= 20 \, pN/nm$, $B_t = 215 \, nN \cdot \mu m^2$

**Table 1:** Values employed for the geometrical and constitutive parameters of the cell equivalent structural scheme

Parameter	Description	Value
$d_i$	integrin spacing	$100 \, nm$ (Cao et al. 2015)
$L_p$	adhesion plaque rest length	up to few $\mu m$ (Cao et al. 2015)
*L*	MT and SF rest length	$10-50 \, \mu m$ (Fraldi et al. 2019)
$A_t$	MT rest cross-sectional area	$190 \, nm^2$ (Fraldi et al. 2019; Deguchi et al. 2006; Pampaloni et al. 2006; Kurachi et al. 1995)
$A_f$	SF rest cross-sectional area	$10^4\, \pi \, nm^2$ (Deguchi et al. 2006)
$k_i$	integrin stiffness	$5 \, pN/nm$ (Cao et al. 2015)
$k_p$	plaque stiffness	$1 \, pN/nm$ (Cao et al. 2015)
$k_n$	nucleus stiffness	$10-50 \, pN/nm$ (Cao et al. 2015)
$k_s$	ECM/substrate stiffness	$1-100 \, pN/nm$ (Cao et al. 2015)
$E_t$	MT Young modulus	$1.2 \, GPa$ (Fraldi et al. 2019)
$E_f$	SF Young modulus	$1.45 \, MPa$ (Deguchi et al. 2006 )
$B_t$	MT bending stiffness	$(10^{-2}-10^{2})2.15 \, nN \cdot \mu m^2$ (Fraldi et al. 2019; Brangwynne et al. 2006)
$\Delta \mu _0$	chemical potential gradient at zero force	$(10-250)\, k_B T$ (Cao et al. 2015)

By numerically determining the latter solution, it is possible to observe that it arises only for values of the inelastic contractile stretch overcoming a critical threshold, namely $\lambda _{f,cr}^c$, which in fact represents an equilibrium bifurcation point for the system’s mechanical response (Timoshenko 1961; Bigoni 2012). The trend of this critical actomyosin contraction is plotted in Fig. 2a as a function of the adhesion plaque size and with reference to the realistic ranges of the parameters’ values resumed in Table 1, while the overall bifurcation diagram of the structure is shown in Fig. 2b. Therein, stable and unstable equilibrium paths are highlighted and obtained as the level of actomyosin contraction grows starting from a resting condition, the stability having been classically studied by evaluating the Hessian matrix of the total potential energy and in particular its minima, according to the Lagrange–Dirichlet theorem. Finally, Fig. 2c and d illustrate how the resulting elastic aliquot $\lambda _f^e$ of the stretch in the SF and the purely elastic stretch $\lambda _t$ in the MT vary along the system’s stable equilibrium path. In particular, since kinematical compatibility has to be guaranteed, the SF turns out to be growingly elongated elastically as the inelastic contraction increases, with $\lambda _f^e$ exhibiting a functional change at the buckling event. As a consequence, this implies that growing tensile forces stand on the SF. On the other hand, the MT elastically contracts in the axial direction during the pre-buckling phase and then it retains a constant contraction level throughout the post-buckling regime in which it assumes an increasingly deviated configuration. It is worth noting that such constant MT contraction level does not depend on the initial size of the adhesion plaque, the latter only determining the speed at which it is achieved. All these results are in accordance with the tensegrity nature of the CSK: they in fact show how the intracellular inelastic contraction produced in the SF by ATP-triggered myosin motors induces elastic deformations and hence tensile stresses, which are balanced by compression in the MT, in addition to the reaction forces of the ECM at the FA. In particular, it can be observed that the balancing role of the MT reduces as it buckles since, in that case, for growing elastic stretch in the SF, the contraction in the MT keeps instead constant. In this situation, the ECM plays hence a prevalent mechanical role, as also confirmed by the outcomes presented in the following sections, which show an amplification of the force level transmitted to the FA-ECM complex when buckling occurs. On the contrary, the tensile force in the SF would be fully balanced by the sole compression in the MT in case of free boundary conditions.

## Influence of mechanics on the growth rate of FAs

It is known that mechanical forces have a significant impact on the polymerization and depolymerization of protein aggregates in cells (Stamenović and Ingber 2009; Shemesh et al. 2005). These phenomena are in fact determined by the tendency of a biopolymer to exchange molecules with the surrounding cytosol, which can be essentially expressed in terms of chemical potential gradient between aggregated particles and free monomers in solution (Hill and Kirschner 1982; Shemesh et al. 2005). By indicating with $\mu _p$ the chemical potential associated to proteins within the polymeric ensemble that is the adhesion plaque in the case at hand (see Fig. 1b and e), the Gibbs–Dühem equation relates an infinitesimal variation of such potential to an infinitesimal change of axial force with respect to a generic current value *F* as follows:

3.1 $$\begin{aligned} d \mu _p = - l_m(F) \, dF =- L_m\lambda (F) \, dF, \end{aligned}$$

where $l_m(F)$ identifies the actual length of the single molecular constituent, related to the rest length $L_m$ through a stretch $\lambda (F)$. It is worth noting that all the variables appearing in the previous equation can be functions of the reference position coordinate, say *X*, therein omitted for the sake of simplicity. Then, under the assumption of linearity made here for mechanically characterizing the adhesion plaque, it is possible to set $\lambda =1+F/E_pA_p$, so that, after integration, it results:

3.2 $$\begin{aligned} \mu _p(X)=\mu _p^0-d_i F(X) -\dfrac{F^2(X)}{2k_p}, \end{aligned}$$

having here considered $L_m=d_i$ and $\mu _p^0$ being the chemical potential characterizing particles belonging to the plaque in absence of mechanical forces. As a consequence, the difference of chemical potentials between bounded and free molecules can be expressed as

3.3 $$\begin{aligned} \Delta \mu (X)=\mu _p(X)-\mu _{free}=\Delta \mu _0-d_i F(X) -\dfrac{F^2(X)}{2k_p}, \end{aligned}$$

$\Delta \mu _0=\mu _p^0-\mu _{free}$ being the chemical potentials difference at vanishing force.

Finally, being $\Delta \mu$ the driving force for the transfer of monomers between polymer and solution, it is realistic to assume the local molecular flux towards the plaque as given by $j(X)=-D \Delta \mu (X)$, with *D* a positive coefficient governing the assembly kinetics. In this way, a negative $\Delta \mu$ induces a recruitment of additional components by the plaque, while a positive $\Delta \mu$ activates local depolymerization. However, the exchange of molecules essentially takes place at the sole extremities, so that the total growth rate of the adhesion plaque can be obtained as

3.4 $$\begin{aligned} J=j(0)+j(L_p)=-D \left[ \Delta \mu (0)+\Delta \mu (L_p)\right] . \end{aligned}$$

Then, by taking into account that the plaque is free at the left end, i.e. $F(0)=0$, and loaded on the right side by the axial force coming from the SF-MT system, say $F(L_p)=F_a$ (see Fig. 1d and e), one finally has:

3.5 $$\begin{aligned} J=-D \left( 2 \Delta \mu _0-d_i F_a -\frac{F_a^2}{2k_p} \right) , \end{aligned}$$

where the force $F_a$, pulling both the FA-ECM complex and the nucleus, can be simply obtained as $F_a=k_{eff} \Delta _{eff}=k_n \Delta _n$ once solved the mechanical equilibrium problem of the cell’s equivalent structure as reported above.

## Actomyosin contraction as active tuner of the FAs’ assembly

By taking into account the kinematical origin of tensile forces in SFs and their mechanical interaction with compressed MTs, the presented approach allows to retrace results previously pointed out in literature, by additionally generalizing them to cases in which large deformations and instability take place.

**Fig. 3:**
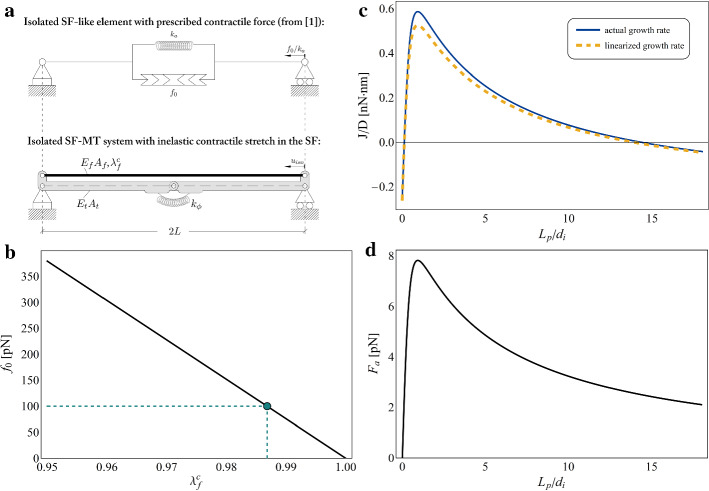
**a** Isolated SF element from the work by Cao et al. (2015)—referred as [1]—(on the top) and the SF-MT tensegrity system extracted from the cell structural description considered in the present work (on the bottom): scheme for the kinematical derivation of the contractile force $f_0$ as a function of the actomyosin contraction level $\lambda _f^c$ through the equivalence of the two models. **b** Variation of $f_0$ in terms of $\lambda _f^c$ and (in green) identification of $\lambda _f^c=0.987$ as corresponding to a contractile force $f_0=100\,pN$ (Cao et al. 2015). **c** Normalized growth rate of the adhesion plaque *J*/*D* and **d** pulling axial force $F_a$, both obtained for $\lambda _f^c=0.987$ at the current configuration in Fig. 1d, as functions of the normalized plaque’s length $L_p/d_i$. All the plots refer to values of the model’s parameters reported in Table 1, by in particular setting: $L=E_f A_f/k_a$, $k_s=10 \, pN/nm$, $k_n= 20 \, pN/nm$, $B_t = 215 \, nN \cdot \mu m^2$, $\Delta \mu _0=30\, k_B T$ and $k_a= 50 \, pN/nm$ (Cao et al. 2015)

To show this, we start by making a direct comparison with findings presented in the reference work by Cao et al. (2015), where an entirely linear and structurally simpler model of adherent cell is considered, by analogously treating the nucleus and the FA-ECM complex as equivalent springs, but by including, for the interconnecting cytoskeletal system, the sole actomyosin network contribution while neglecting the presence of MTs. In particular, to make the comparison, let us consider the mechanical scheme shown on the top of Fig. 3a, corresponding to the actomyosin SF element utilized by Cao et al. (2015) when isolated from its elastic surroundings (i.e. the nucleus and the FA-ECM complex). Therein, a contractile element translates the effect of myosin motors in the elastic SF by prescribing an axial force $f_0$ at the ends of a linear spring of stiffness $k_a$, this in turn entailing an axial contractile displacement of magnitude $f_0/k_a$. On the other hand, by examining the SF-MT system on the bottom of the same figure, extracted from the cell mechanical model employed in the present work, in which the origin of cytoskeletal forces is ascribed to a myosin motor-guided kinematics, one can derive that the activation of an inelastic contraction $\lambda _f^c$ within the SF produces an overall axial displacement equal to

4.1 $$\begin{aligned} u_{iso}=L\left\{ 1-\left[ \dfrac{E_t A_t \lambda _f^c + E_f A_f \left( \lambda _f^c \right) ^3}{E_t A_t \lambda _f^c +E_f A_f}\right] ^{1/3}\right\} \end{aligned}$$

before buckling takes place. Therefore, by requiring the equivalence between the displacements undergone by the two structures, the following relationship can be obtained:

4.2 $$\begin{aligned} f_0= E_f A_f \left\{ 1-\left[ \dfrac{E_t A_t \lambda _f^c + E_f A_f \left( \lambda _f^c \right) ^3}{E_t A_t \lambda _f^c +E_f A_f}\right] ^{1/3}\right\} , \end{aligned}$$

which links the value of the contractile force $f_0$, *a priori* prescribed by Cao et al. (2015), to the actual level of actomyosin contraction, as shown in Fig. 3b, thus restoring the kinematical nature of the intracellular forces. In particular, in this way, it can be found that a contractile force of magnitude $f_0=100 \, pN$, as that considered in the work by Cao et al. (2015), can be obtained by rather imposing a kinematical contraction $\lambda _f^c=0.987$. Note that, in Eq. (4.2), it has been additionally set $L=E_f A_f/k_a$ in order to guarantee the correspondence of the elastic and geometrical properties employed for the SF in the two models.

Then, by assuming that the SF belonging to the assembled cell model proposed in this work and sketched in Fig. 1 withstands an inelastic stretch $\lambda _f^c=0.987$ and by adopting the mechanical and thermodynamic strategies described in the previous sections, it is possible to recover the response of the overall system in terms of growth rate and related pulling axial force at different stages of the adhesion plaque size, as shown in Fig. 3c and d. In this way, for the selected magnitude of inelastic contraction, the length of the adhesion plaque determines, in accordance with the axial force level, the direction and magnitude of the monomers flow: in particular, two different regimes can be experienced by the plaque, that is a polymerization phase (identified by $J>0$) at intermediate lengths, promoting adhesion stabilization, and a depolymerization state (i.e. $J<0$), affecting both FAs below a minimum nucleation size and anchorage sites beyond a certain critical length. This outcome confirms what highlighted in the work by Cao et al. (2015), where the Authors find that the FA growth rate is positive only when the plaque’s length ranges between a lower critical value that a nascent adhesion site must overcome to initiate elongation and an upper stable size over which disassembling takes place. Indeed, newly nucleated focal complexes with sizes smaller than the critical one result unstable and dismantle, while excessively large FAs are predicted to shrink up to reach the maximum stable length. With reference to Fig. 3c, it is also worth highlighting that, in accordance with the observation that the hypothesis of small deformations holds true for the considered level of inelastic contraction, the curve obtained by considering the expression of the growth rate in Eq. (3.5)—i.e. the blue solid one—turns out to be very close to that found by neglecting the second-order term and by considering $J \approx -D \left( 2 \Delta \mu _0-d_i F_a \right)$ as *a priori* assumed by Cao et al. (2015), i.e. the yellow dashed curve. However, this approximation would not generally hold true for arbitrarily higher deformations as those that could also occur in real cases, for this reason considered in the present work.

**Fig. 4:**
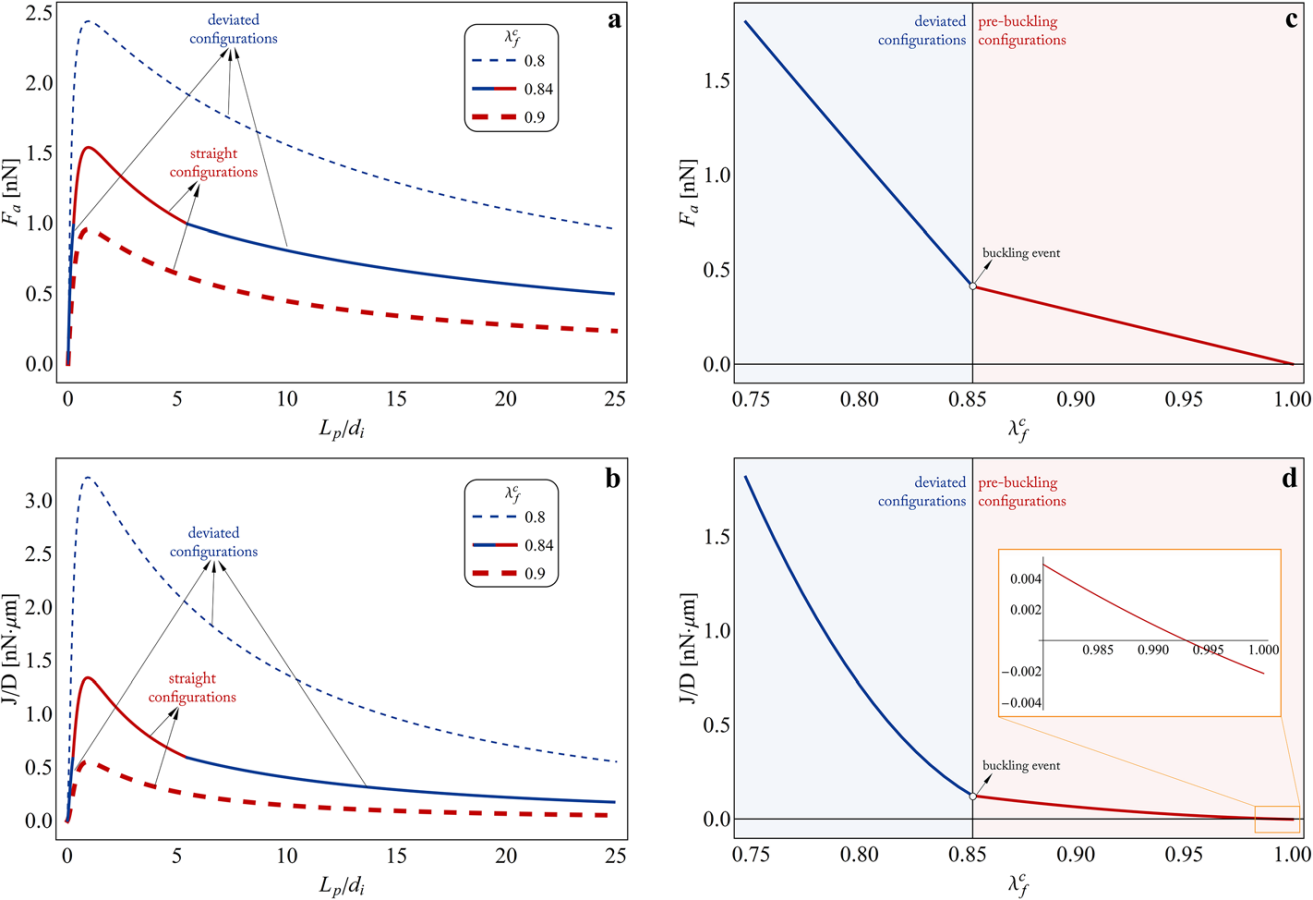
**a** Pulling axial force $F_a$ and **b** normalized growth rate *J*/*D* plotted as functions of the adhesion plaque length $L_p$ normalized with respect to the integrin spacing $d_i$. The curves refer to three different values of inelastic contraction such to keep the MT straight ($\lambda _f^c= 0.9$: red dashed curve), to induce MT buckling independently from the plaque length ($\lambda _f^c= 0.8$: blue dashed curve) or to cause instability if outside a certain range of FA size ($\lambda _f^c= 0.84$: solid curve, red-coloured for the straight configurations and blue-coloured for the deviated states). **c** Pulling axial force $F_a$ and **d** normalized growth rate *J*/*D* plotted as functions of the actomyosin contraction level occurring in the SF before (red tracts) and after (blue tracts) buckling of the MT, for a fixed magnitude of the plaque length $L_p=20 \,d_i$. All the plots refer to values of the model’s parameters reported in Table 1, by in particular setting: $L=20\, \mu m$, $k_s=10 \, pN/nm$, $k_n= 20 \, pN/nm$, $B_t = 215 \, nN \cdot \mu m^2$, $\Delta \mu _0=250\, k_B T$

An extension of the previous outcomes is for instance provided in Fig. 4, where the effects of increasing levels of actomyosin contraction on the FA growth rate are analysed in detail, since the contractile activity occurring in the SF can vary depending on the number of moving myosin heads triggered via ATP hydrolysis as well as on the magnitude of the resulting inelastic sliding among pairs of actin filaments. Specifically, as shown in Fig. 4a and c, a growing contractility degree can determine a significant increase of the value of mechanical force pulling on the FA-ECM complex with respect to those presented above, with a further abrupt amplification occurring in correspondence of the MT buckling event at the critical contraction threshold. This mechanism in turn powers and regulates the processes of polymerization and depolimerization of the FA by coherently enhancing the flux of proteins binding to the plaque, as reported in Fig. 4b and d. By looking in particular at the inset in Fig. 4d, it is also possible to observe how, at fixed length of the adhesion plaque, contractility levels higher than a critical threshold have to be necessarily reached in order to promote monomers aggregation, plaque disassembling associated to negative molecular flux in fact taking place otherwise. These results, confirming and generalizing observations in part outlined in literature (e.g. by Cao et al. (2015) and Shemesh et al. (2005)), shed light on the fact that size is not the only element governing the growth/dismantling tendency of the FA, the magnitude of actomyosin contraction playing an essential role in both triggering and modulating polymerization and depolymerization events. Furthermore, differently from the adhesion plaque’s starting length, the level of actomyosin contraction represents a parameter that cells can actively and continuously modulate by essentially promoting or inhibiting energy (ATP) consumption to trigger myosin motor-mediated sliding in SFs. This hence allows to show how biochemically or mechanically induced active tuning of actomyosin contraction represents the main mechanism that cells could adopt to start, to control and to halt local as well as diffused rearrangements of their anchoring loci, which are prodromal to the remodelling undergone by the intracellular architecture during cell adhesion/detachment, migration and reorientation activities.

**Fig. 5:**
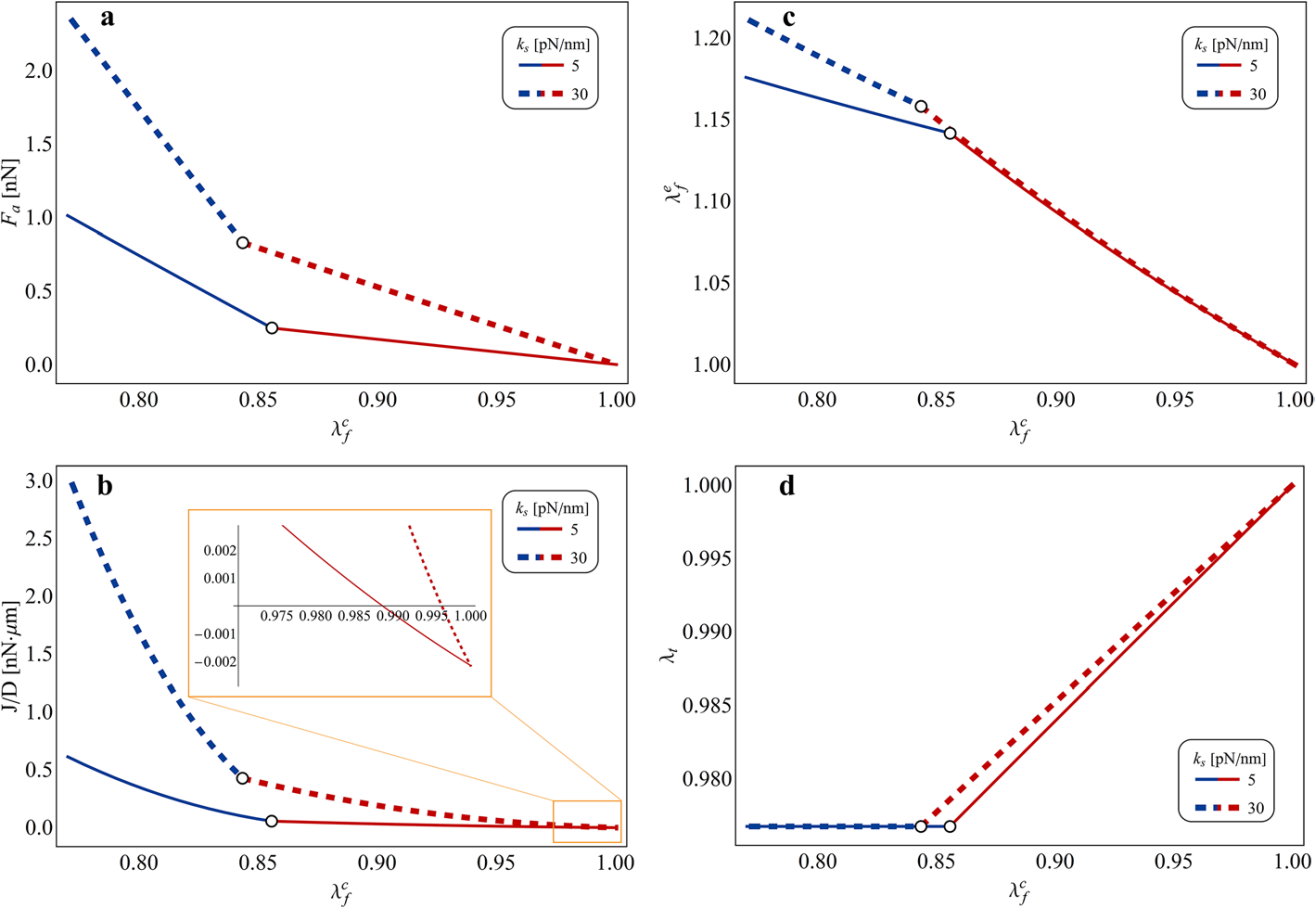
**a** Pulling axial force $F_a$, **b** normalized growth rate *J*/*D*, **c** elastic stretch aliquot in the SF $\lambda _f^e$ and **d** stretch in the MT $\lambda _t$, all plotted for varying actomyosin contractile stretch $\lambda _f^c$ at two different values of the ECM stiffness, i.e $k_s=5\, pN/nm$ and $k_s=30\, pN/nm$, compatible with ranges measured in healthy and tumour environments, respectively. The colours red and blue are adopted for indicating curves’ tracts related to pre-buckling and post-buckling configurations, respectively. All the plots refer to values of the model’s parameters reported in Table 1, by in particular setting: $L=20\, \mu m$, $L_p=20 \, d_i$, $k_n= 20 \, pN/nm$, $B_t = 215 \, nN \cdot \mu m^2$, $\Delta \mu _0=250\, k_B T$

Finally, it is worth highlighting the effects determined by different rigidities of the adhesion medium on the elastic deformations and the stresses arising within the cell and, as a consequence, on the molecular dynamics of the FAs, as a result of the contractile activity developing in SFs. In this regard, Fig. 5a and b mark how the stiffening of the ECM, for fixed levels of actomyosin inelastic contractile stretch, leads to the increase of the pulling force acting on the FA-ECM system and, hence, of the assembling rate of the adhesion plaque. Moreover, as enlightened in the inset of Fig. 5b, a greater substrate’s stiffness lowers the minimum threshold of actomyosin contraction that has to be reached in the SF in order to halt monomers disassembling and to rather promote FA polymerization. These considerations are in accordance with predictions from other literature theoretical models (Cao et al. 2015; Ronan et al. 2014; Vernerey and Farsad 2011) as well as with experimental results, which show that the building up of FAs is facilitated over stiffer substrates, where in fact they occupy larger areas with respect to sizes observed in case of soft materials and where cell adhesion turns out to be more stable (Prager-Khoutorsky et al. 2011; Trichet et al. 2012; Fusco et al. 2017). On this basis, cells’ predilection for stiffer substrates, which leads for instance to the so-called *durotaxis* phenomenon (Lo et al. 2000; Trichet et al. 2012), can be read as an optimization mechanism carried out by cells to enhance their adhesion capabilities without increasing the biochemical energy expense devoted to actively generating SFs’ inelastic contractions. In accordance with this logic, it is also possible to observe in Fig. 5c and d that the ECM stiffness increase leaves almost unchanged the magnitudes of the elastic stretches in both the SF and the MT, at least in the pre-buckling phase. This implies that a stiffer ECM allows the cell to pull with higher mechanical forces on the FA-ECM ensemble—and thus to rise the FA growth rate—without involving any increase of the elastic energy stored in the cytoskeletal apparatus. In particular, the latter observation could also help to gain some insights in the reason why tumour cells generally exhibit reduced stiffness and lower adhesion degree, with smaller and more dynamic FAs, associated to a stiffer surrounding ECM with respect to the healthy counterpart (Fraldi et al. 2021, 2015; Nebuloni et al. 2016; Cross et al. 2007; Goetz et al. 2011). In fact, to be anchored to a stiffer environment would give to cancer cells the possibility to lower the level of actomyosin contractility required to build up FAs—which are however necessary to accomplish migration—but, at the same time, the combination of the reduced actomyosin contraction with the result that the ECM stiffness increase does not make intracellular stresses rise, would also provide them with a smaller overall stiffness (Fraldi et al. 2019).

## Conclusions

The present work focuses on the bio-mechanical modelling of an adherent single-cell for analysing the way in which, by actively modulating the level of actomyosin contraction in SFs, the cells are able to control and to continuously re-adapt the configuration of their protein anchoring sites to the ECM that is the FAs. More in detail, the aim is contributing to investigate the role of actomyosin contractility in FAs’ growth and disassembly by generalizing consolidated literature approaches (e.g. the one adopted by Cao et al. (2015)) by taking into account—for the first time—relevant and previously neglected mechanical aspects, such as the tensegrity-like distribution of forces across the CSK, the nonlinear elasticity of the cytoskeletal filaments and the elastic instability observed in MTs *in vivo*. The cell is in fact seen as an essential (minimal) structural ensemble comprising a linear elastic nucleus that binds to a spring-like FA-ECM network through a cytoskeletal complex. The latter consists of a SF-like unit bounded in parallel with a MT-like element, thus providing the simplest paradigm of tensegrity system, giving self-equilibrium of the nonzero pre-stress states in absence of external forces. To take into account possible large deformations and the nonlinear elastic response of the cytoskeletal biopolymers, both the SF and the MT are modelled as hyperelastic by adopting a standard neo-Hookean constitutive law. Furthermore, to trace phenomena of buckling generally observed in MTs of living cells, the MT-like component is equipped with a nonlinear elastic hinge allowing it to assume deviated (i.e. folded rather than purely axially deformed) states at some critical compression levels.

Furthermore, a key novelty point of the work is that of providing a different, more faithful, mechanical interpretation of the intracellular contractile forces with respect to standard modelling strategies, by transducing their origin, ascribed to a chemically activated kinematics carried out by myosin motors within SFs, into an inelastic deformation aliquot, as it actually is. By indeed starting from a stress-free reference configuration and in absence of externally prescribed loads, elastic deformations and stresses are all induced on the cell’s constituents by an ATP-driven intracellular actomyosin contraction, explicitly described as an inelastic stretch arising inside the SF-like unit, which in turn entails the elastic counterpart to ensure geometrical compatibility.

Overall, by introducing the new mechanical elements described above and by following a classical thermodynamic approach to relate the growth rate of the cell’s FA unit to the elastic force pulling on it, we show how our model provides a generalized and consistent strategy to predict some nonlinear phenomena characterizing the mechanobiology of the cell’s complex, even when some simplifying modelling assumptions have to be necessarily removed. This also leads to recover, under limit benchmark conditions, outcomes previously presented in literature, according to which the initial size of the adhesion plaque and the stiffness of the anchoring substrate tightly orient the molecular dynamics of the FAs. In particular, the theoretical results highlighted the pivotal function assumed in this process by the actomyosin contraction occurring in SFs, by mainly investigating the effects of growing contractility levels and of related elastic instability phenomena, on the tendency of the FAs to polymerize and to depolymerize.

It is felt that the proposed theoretical formulation could contribute in defining effective analysis strategies for the modelling of bio-chemo-mechanical processes occurring within living cells, which could be for example enriched thanks to the possibility both of taking into account a more complex cytoskeletal apparatus, starting from the synergistic effect of bundles of biopolymers aligned along one direction, and of involving molecular assembling/dismantling events in both FAs and CSK biofilaments, from those leading to the structural reconfiguration of stable adherent cells in response to external mechanical inputs or to intracellular signals up to those implicated in chemically or mechanically induced mechanisms of cell locomotion. Also, modelling and predicting of alterations of these phenomena could help to interpret some still unexplained observations related to physiological as well as pathological conditions that involve the mechanobiology of cancer cells.

## Supplementary Information

Below is the link to the electronic supplementary material.

Supplementary file1 (AUX 1 kb)

Supplementary file1 (LOG 40 kb)

Supplementary file1 (AUX 20 kb)

Supplementary file1 (BLG 1 kb)

Supplementary file1 (OUT 1 kb)
